# News and Miscellany

**Published:** 1885-04

**Authors:** 


### NEWS AND MISCELLANY.

Personal.—Dr. William Osler of McGill University and Veterinary College, Montreal, has received a unanimous invitation to the chair of Pathology in the University of Pennsylvania.

W. L. Zuill, D.V.S., of Philadelphia, has been selected to fill the chair of surgery in the Veterinary Department of the University of Pennsylvania.

William Herbert Lowe, D.V.S., has been appointed by Dr. Hunt of the New Jersey State Board of Health one of the State Veterinary Inspectors.

---

Hog-Cholera.—Dr. A. A. Holcombe, State Veterinary Surgeon of Kansas, has written a special report upon the nature, cause, prevention, and treatment of hog-cholera, which has been published by the State Board of Agriculture, for distribution among the people. Copies can be obtained by addressing Hon. Wm. Sims, Secretary, at Topeka, Kansas.

---

Brain of Monkey.—There is in the collection of Dr. E. C. Spitzka of this city, the brain of an Entellus monkey (*Semnopithecus entellus*) which had died in the Central Park Zoological Gardens two winters ago, which is interesting owing to the presence of a large cysticercus in the cortex bordering below on the second temporal sulcus. No symptoms were connected with this, as far as ascertainable, death being due to other causes.

---

Dog Meat as food for Man.—According to the "Veterinary Medical Annales," the Professors of the Veterinary Medical College of Brussels answered the inquiry of the Minister of the Interior, "Is there a reason why dog-meat should not be used as food for man?" The sale of it is now permitted, on an inspection certificate of an authorized veterinary surgeon.

The following are the points for the guidance of the inspector:

1. Lean dogs, or those having symptoms or lesions of serious diseases, such as rabies or even a suspicion of it, rhachitis, dropsy, abscess, tumors of any kind, intestinal, hepatic, peritoneal inflammation, pneumonia, pleuritis, are excluded.

2. Pharynx, stomach and intestines are under all circumstances excluded from sale.

The sale is limited to *one* butcher's-stall, with a sign of "Dog-Meat" for sale.

Only the carcases of dogs that had been slaughtered, or whose death was caused by the loss of blood from a divided jugular, can be offered for sale.

It is not known whether the Belgians have an especial taste for dog-meat; or is the dearth of meat-food so great that this extreme measure must be resorted to? However, *De gustibus non est disputandum.*

---

NATIONAL VETERINARY ASSOCIATION.—The following officers have been elected to serve for the following year: President, Dr. L. V. Plageman, New York; Vice-Presidents, W. C. Fair, Ohio; William Sheppard, Illinois; J. W. Salladay, Penn.; Corresponding Secretary, W. T. Carmody, New York; Recording Secretary, R. Hardy, Illinois; Treasurer, A. J. Chandler, Michigan.

---

SELF-MUTILATION IN A LIONESS.—Dr. P. S. Abraham in the *Dublin Journal of Medical Science* states:

On the 18th of May, last year, a fine lioness in the Zoological Gardens, in Dublin, was discovered to have devoured, during the night, some six inches of her tail—the hair, skin, bones, and everything. She did not then touch it for some days, but appeared to be very restless, and on the 27th of the month she recommenced her extraordinary conduct, and demolished, during the night, the greater part of the remainder of the organ. She then rested awhile, but again went at it, and in four weeks from the time she began there was nothing left of her caudal appendage but the "butt"—some four inches or so. The organ was now so short that she could not reach it with her mouth, and it was hoped that in consequence she would resume her usual habit, and be satisfied with the flesh of other animals; but on the 1st of July she began to lick and gnaw off the skin of the dorsum of the right hind paw. The integument and subjacent tissue are seen to be removed from nearly the whole of the extensor surface of the foot, and it is evident that the tendons would be exposed were it not for the granulation tissue which has formed, as a superficial layer, over them. It was quite certain that while all this was going on the animal suffered extreme pain; the stump of the tail was to be seen in a constant state of quiver, and when the back of the foot was gone the leg was drawn up, and the creature limped about the cage on her three legs.

There was nothing apparently to account for this strange behavior on the part of the lioness. She was in splendid condition as regards the fur, flesh, and appetite, and the excretions were normal. It is needless to say various methods were tried to induce her to leave herself alone—complete change of food, sulphur and other aperients, worm-powders, syringing the parts with bitter liquids, &c.—but all with no effect. At last, indeed, it was deemed advisable to destroy her, for her suffering seemed so great, and the extent of the wound on the foot was so large, that, even if she left off eating it, it appeared impossible that it could ever properly heal and skin over.

At an examination of the body, made shortly after death, I found the thoracic and abdominal organs all perfectly normal; the right ovary was larger than the left, and its surface presented several large protrudent Graafian follicles. I was at first inclined to think that this ovary showed considerable abnormality, but after consultation with Dr. Neville, and on its microscopical examination, I think it probable that, beyond some degeneration, it is the seat of no very great pathological change. The brain and spinal cord were not examined.

The lioness, who was about twelve years old, had been in the gardens for five years, and had always been in good health. She had produced cubs three times, but her offspring were, with few exceptions, unhealthy, mostly becoming rickety and dying young. For one year previous to May, 1884, she had not been in season, although formerly she had been tolerably regular in "coming round."

It is well known that foxes and many other animals when trapped by one foot will sometimes gnaw themselves free, and leave a portion of their persons behind; and a gradual gnawing and picking away of the tail has been observed as a not uncommon habit with monkeys in confinement, as well as occasionally in dogs, rats, and some other creatures. The present case, however, does not come under precisely the same category as these, for there appeared to be absolutely no external cause for the procedure, and instead of a gradual gnawing away and disappearance of the organ, large pieces were scrunched off at intervals and swallowed.

I have made a great many inquiries as to similar occurrences in other zoological gardens and menageries, and, on the whole, I am inclined to consider that this departure from the creature's usual habit is due rather to something akin to mental derangement. My inquiries tend to show that the carnivores which have "taken on" in this way have been nearly always females, which have either been very young, just before they began to breed, or old, at the menopause, when their breeding period had come to an end—at any rate, that there has always been some interruption or disturbance of the sexual function; and I venture to suggest that we may look upon this perversion of taste, in our lioness at all events, as one of the manifestations in the lower animals of that protean affection which we call "hysteria.'

---

Sir Astley Cooper and his Operations on Animals.—When Sir Astley was rather old he purchased a splendid country seat near London. As long as he could find a case of disease in his horses, cows, sheep or pigs, he was delighted and attended them with all the interest and fidelity he would have shown to a human being, and often trepanned the head of some favorite ram or ewe in search of the cause of its disease in the head. But the moment he found his stock in perfect condition he at once became restless and unhappy, and sighed for the wards of Guy's Hospital. J. C. P.

---

Velpeau's early Career.—Velpeau was the son of a blacksmith in the village of Breches in the province of Loire. His father's library consisted of two books, one the Complete Drover, the other a volume of medical receipts. He learned the latter by heart, and turned his attention to medicine. He went twice a week to a country school 3 miles off, but continued to shoe horses and prescribe for their diseases until his 23d year, although he devoted many hours of the night to improving his mind. Then he set out on foot for Paris, but was obliged to stop at Tours to replenish his purse by horse-shoeing. Arrived at Paris he hired a black coat for 3 francs, which did not fit and contrasted strangely with his country garments. Thus attired he called upon the celebrated Dr. Dubois, offering to become his pupil, who notwithstanding his long-tailed coat and sky-blue pantaloons told him he might have the run of his kitchen until he could ascertain his talents and qualifications. He was soon regarded as a companion and friend by Dubois, as he made astonishing progress in his medical studies and was so diligent and untiring as to acquire in a short time such knowledge of the classics and most modern lauguages of Europe, including English, as to read them with facility. He was so careless of his dress that an assistant in a watchmaker's shop who was his native friend was requested to keep special watch over him, as he was regarded as a suspicious character, a might-be thief. J. C. P.

---

N. Y. ACADEMY OF VETERINARY SCIENCE AND COMPARATIVE PATHOLOGY. —A number of veterinary surgeons, residents of New York and its vicinity, assembled in the United States Hotel March 24, and organized what is to be known as the New York State Academy of Veterinary Science and Comparative Pathology. Among those present were Prof. Peter Peters, Prof. James Hamill, Dr. Charles A. Meyer, Dr. Harry D. Gill, and Dr. Thomas Robertson of this city; Dr. Henry E. Early, of Richmond, Staten Island; Dr. L. V. Plageman, Brooklyn, and Dr. William T. Middleton, of Fishkill, N. Y. Prof. Hamill said the purpose of their organization was to bring forward a society of independent surgeons for the promotion of veterinary science and mutual protection. He said 40 years ago an organization of this kind was perfected, but it died out for lack of enthusiasm. Another was formed in October, 1883, but this fell through by the treachery of a clique who wanted to further their own interests. He hoped that nothing of the kind would befall the present society, and he asked his colleagues to leave nothing undone to bring it up to the highest standard. Incorporation papers were drawn up and signed and the meeting adjourned.

---

THE CUNNING OF THE OPOSSUM.—The cunning of the opossum has been repeatedly called into question; the following remarkable facts, however, suggest the necessity for further observation, before committing ourselves to the absolute denial, which a recent distinguished writer has made. Dr. E. C. Spitzka had a living opossum presented to him in 1879 which he kept alive for some days, before using it for anatomical investigation. It was confined in an oblong box, about thirty inches long, twelve wide, and as many high. This was of wooden boards on all sides except the top, across which slats of wood, were nailed at intervals of not quite an inch. The animal ate greedily in confinement. It was the custom every night to lay a heavy object—on the occasion mentioned a stone—on the top of the cage, as the box was of rickety make, and it was thought that the middle slats could have been forced upwards. One morning, the owner came down to the basement, to which no person but himself had access, and found the stone *nearly in its usual place*, the *slats underneath it gnawed through from the inner side* to the number of three, *but replaced as to direction.* The only explanation is, that the animal had shoved the stone to one side, had gnawed through the bars, gone out, *and returned to its prison,* pulling the bars in place again, and working the stone nearly back to its former place, or that some one had played a joke on the owner, with the purpose of mystifying him—the latter alternative is to be rejected, as it would have necessitated a combination of circumstances scarcely possible. The doors and windows were locked. Jars in which stychninized frogs were confined had been overturned; and much confusion created among the paraphernalia of the laboratory; several of the animals had been devoured, for careful search did not reveal any trace of them, and it was believed that the opossum had eaten them in his nocturnal ramble. The frogs mentioned were kept in chronic stychnine tetanus by mineral doses of acetate of stychnine, insufficient to have affected the opossum.

If the view above expressed be correct, it would show the possession on the part of the opossum of a low kind of cunning in the same direction as its alleged simulation of death. The animal showed skill in escaping, stupidity in returning to prison, and some method in concealing the traces of its escape.

Whether (under this theory) it returned to its prison from sheer habit—having become accustomed to that habitation—or because it associated the idea of abundant food with it, is a question that cannot be answered. It is, certainly, one of the most singular *appearing* set of puzzling facts that has come to the notice of one who has had frequent opportunities of studying the habits of animals kept in captivity. That it deliberately intended to produce deceptive appearances is not supposed, but rather that it replaced the objects in their former position, in obedience to an instinctive tendency to concealment during the day—as it is a nocturnal animal.

---

A Monstrosity.—In the spring of 1881, while in Southern Indiana, the Rev. F. M. Symmes, of Paoli, Ind., brought to my office a lamb or lambs, with the following anatomical structure: The integument from near the middle of the bellies toward and including the hinder extremities was as with separate lambs. From a point just in front of the umbilici the integument was joined and enveloped a double thorax. There are four legs which are in such a position that the legs of each embrace or pass by the other. The two necks were also enveloped by the same skin. The head, having but one mouth, would hardly be called double. The mouth is turned in a direction between the two bodies. The head had four ears, two at the sides and a little anterior to their normal position; and two small ears at the back of the head, joined at the base.

There were two eyes, one a large one, located directly over or back of the nose. It had a very deep and oblong orbit. It had the appearance of having a double pupil, but upon dissection, there was found but a single opening through the iris, with a white perpendicular line in the cornea. The eye was supplied with a single optic nerve which branched at the commissure and had a right and left optic tract. The other, a smaller eye, was at the back of the head, and immediately above the little double ears. This eye had but a rudimentary optic nerve, which was not developed of sufficient length to reach the brain. The orbit of the little eye was composed only of a bony disc which had but a slight connection with the cranial bones.

There is but a single brain cavity. The lateral diameter of the head is the greater; especially the base is wide, where it is attached to the vertebræ. The cervical vertebræ are parallel and face each other. At the beginning of the dorsal section each column makes an acute curve backward; then a more gradual curve forward until the lower dorsal and lumbar vertebræ are again brought parallel These curves to the spinal column give greater expansion and capacity to the single thorax containing a double set of viscera. The ribs of each join on the sternum opposite their fellows from the other vertebral column, thus bringing the two sterni at the sides rather than in front of the vertebræ. On one side, from the fourth to the eighth ribs inclusive, the ribs are joined in one flat, thin bone.

The peculiarities of the viscera, so far as observed, were as follows: There were two larynges, yet each imperfect, with a single pharynx situated between them. The epiglottis of each larynx was normal. The glottides and tracheæ were connected with the œsophagus. The cartilages of the tracheæ were arched in shape and formed lateral segments to the single œsophagus to near half the length of the œsophagus where each trachea separated from the œsophagus forming two bronchi, leading to the lungs on either side. The lungs were separate, forming two right and two left lungs complete. The œsophagus divided at a point just above the diaphragm, one branch going to each of two separate stomachs. All the abdominal viscera were perfect, separate and complete. There were two separate hearts, perfect excepting the exits and entries of the arteries and veins, which were double. There were two aortæ rising from each heart, one branch from each forming a perfect arch with its fellow from the other heart. The other aorta from each heart, being in the normal position of the arch, continued as the thoracic and abdominal aorta, each with its regular branches. The venous arrangement was quite similar and needs no further description.—*D. D. Rose, M.D., in the Weekly Medical Review.*

